# The influence of bentonite content on the performance characteristics of magnetorheological fluids

**DOI:** 10.1039/d5ra09063f

**Published:** 2026-01-30

**Authors:** Shengjie Li, Hu Deng, Junyu Chen, Min Tang, Jian Wang, Ruifeng Jiang, Tao Lin, Weijie Lv, Minxian Lu, Yang Yu, Guang Zhang

**Affiliations:** a School of Mechanical and Electrical Engineering and Transportation, Shaoxing Vocational and Technical College Shaoxing 312000 China; b State Key Laboratory of Fluid Power and Mechatronic Systems, Zhejiang University Hangzhou 310027 China guangzhang@zjut.edu.cn; c XGM Corporation Limited Taizhou 317100 China; d School of Mechanical Engineering, Nanjing University of Science and Technology Nanjing 210094 China; e Institute of Taizhou, Zhejiang University of Technology Taizhou 318001 China; f College of Mechanical Engineering, Zhejiang University of Technology Hangzhou 310014 China; g Zhejiang Zuansheng Technology Co., Ltd Lishui 323000 China; h School of Civil and Environmental Engineering, University of New South Wales Sydney 2052 Australia yang.yu@uts.edu.au; i Taizhou Jinyu Mechanical and Electrical Co., Ltd Taizhou 317100 China; j Zhejiang Hongxin Technology Co., Ltd Taizhou 318020 China; k Key Laboratory of Special Purpose Equipment and Advanced Processing Technology, Ministry of Education, Zhejiang University of Technology Hangzhou 310014 China; l Zhejiang Key Laboratory of High-Precision and Efficiency HybridProcessing Technology and Equipment, Zhejiang University of Technology Hangzhou 310014 China

## Abstract

This study investigates the influence of bentonite concentration (0–2.0 wt%) on the properties of bentonite-modified magnetorheological fluids (BMRFs). The results demonstrate that bentonite significantly enhances sedimentation stability by forming a three-dimensional network structure, with the 1.2 wt% sample exhibiting optimal performance. However, a high concentration of 2.0 wt% led to a complete loss of fluidity, while at 1.6 wt%, partial caking was observed. Rheological tests confirmed that bentonite addition improves the magnetorheological effect, promotes earlier magnetic saturation, and expands the linear viscoelastic region. Furthermore, redispersibility evaluation indicated that the addition of bentonite enabled the BMRFs not only to recover but also to improve their original performance after redispersion, with increases in both apparent viscosity and storage modulus. The 1.2 wt% sample, in particular, demonstrated stronger stability. In contrast, the 2.0 wt% sample exhibited irreversible property degradation. This study provides a valuable formulation for developing high-performance MRFs with balanced stability and redispersibility.

## Abstract 2

This study investigates the influence of bentonite concentration (0–2.0 wt%) on the properties of bentonite-modified magnetorheological fluids (BMRFs).

## Graphical abstract

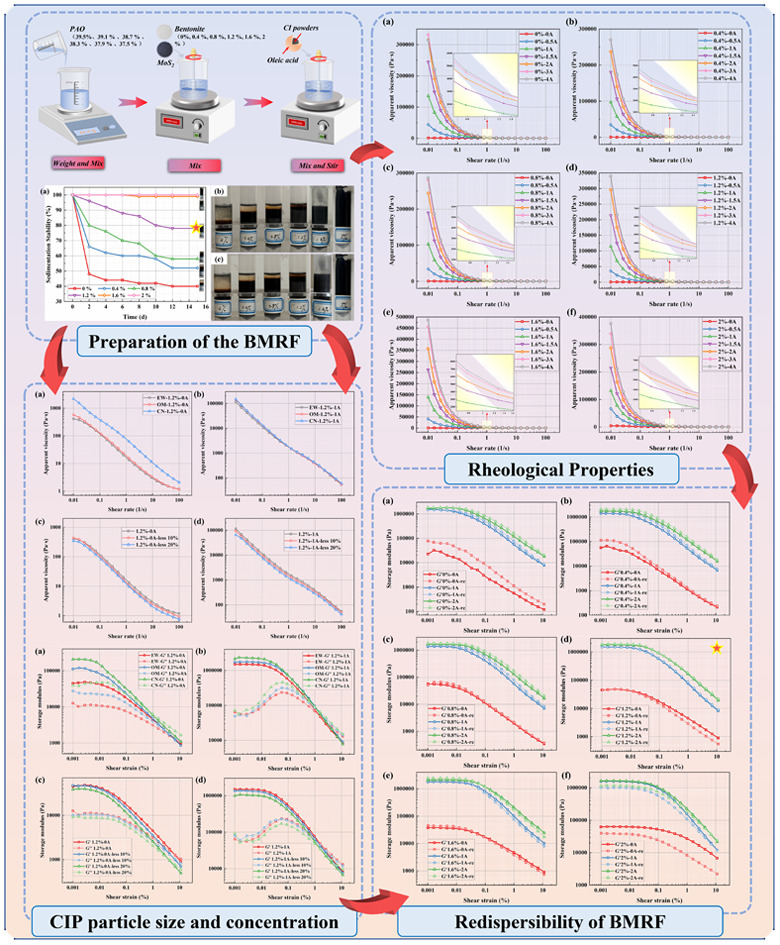

## 1. Introduction

Magnetorheological fluids (MRFs) are a class of smart materials with promising application prospects, characterized by their unique ability to undergo rapid and reversible transitions in rheological behavior upon the application of an external magnetic field—shifting from a near-Newtonian fluid state to a solid-like state with a distinct yield stress.^1,2^ This remarkable field-responsive characteristic enables MRFs to demonstrate significant potential in various fields, including automotive suspension systems,^3^ dampers,^4,5^ controllable clutches,^6,7^ precision polishing,^8^ and wearable rehabilitation training devices.^9^ A typical MRF is composed of three fundamental components: a carrier fluid, soft magnetic particles with high permeability, and additives aimed at improving particle dispersion stability and sedimentation resistance.^10^

Despite these advantages, MRFs face a long-standing technical challenge in practical applications: the significant density mismatch between magnetic particles and the carrier fluid causes particles to inevitably sediment over time, leading to component separation and functional degradation.^11^ This sedimentation results in the formation of a dense sediment cake, which compromises the rheological performance of MRFs—particularly their redispersibility in the absence of a magnetic field—thereby limiting their cyclic service life and long-term reliability.^12^ To address this issue, researchers have adopted various strategies, such as modifying the morphology of magnetic particles,^13^ adding surfactants,^14^ and incorporating thixotropic additives.^15^

Surfactants function by adsorbing onto particle surfaces, generating steric hindrance or electrostatic repulsion to prevent particle agglomeration. In contrast, thixotropic additives form a three-dimensional network structure within the carrier fluid, imparting a certain yield stress to the fluid and thereby effectively hindering particle sedimentation. Meanwhile, bentonite, as a natural, economical, and environmentally friendly layered silicate mineral, has been extensively studied in recent years as a rheological modifier in MRFs.^16^ Zhang *et al.*^17^ tested and analyzed the effects of additives such as stearic acid, sodium dodecyl sulfate, and their mixtures on the rheological and sedimentation properties of magnetorheological fluids. The results indicated that at the same mass fraction of carbonyl iron particles and additives, the shear stress of MRFs with stearic acid was greater than that with sodium dodecyl sulfate. Maurya *et al.*^18^ introduced iron ore as an additive into aqueous magnetorheological suspensions, forming a clay-like gel structure that enhanced the sedimentation stability of the MRFs. Dong *et al.*^19,20^ prepared iron ore/sepiolite nanocomposite particles and incorporated them into carbonyl iron-based magnetorheological fluids, improving the fluid's dispersion and magnetorheological performance. Qian *et al.*^21^ investigated the effects of four clay minerals (sepiolite, kaolin, bentonite, and attapulgite) on the properties of lithium aluminum mixed soap-based magnetorheological greases. The results demonstrated that adding small amounts of clay minerals could enhance the sedimentation stability of the greases, particularly sepiolite, a non-metallic clay with a unique pore structure and high specific surface area. Ren *et al.*^22^ prepared six composite lithium-based greases with different thickeners and studied their rheological properties. The findings revealed that the addition of boric acid introduced coordination bonds, improving the thixotropy and yield stress of the lithium-based magnetorheological greases. Cheng^23^ utilized nano-halloysite to prepare MRFs with different loading energies, demonstrating that halloysite significantly influenced both the sedimentation rate and the magnetorheological performance of the fluids.

However, the addition of bentonite presents a double-edged sword. Empirical observations indicate that an appropriate amount of bentonite can significantly enhance the sedimentation stability and rheological properties of MRFs, whereas excessive addition may lead to an excessively high system viscosity, loss of fluidity, and transformation into a paste-like solid, thereby compromising its applicability in magnetorheological devices requiring rapid response. Furthermore, the influence of the bentonite-formed network structure on the response behavior of MRFs under external magnetic fields—including the magnetorheological effect, magnetic saturation characteristics, and dynamic viscoelasticity—requires more in-depth and systematic investigation.

Previous studies^24,25^ have shown that high-viscosity natural oils, such as castor oil, typically yield higher shear yield stresses and better inherent sedimentation stability than low-viscosity synthetic oils. Although silicone oil and mineral oil are widely used in MRF formulations, PAO oil, compared to high-viscosity fluids, often leads to lower inherent sedimentation stability but offers clear advantages in chemical consistency, a wide operating temperature range, and oxidation resistance. While PAO is susceptible to sedimentation due to its low zero-field viscosity, we have successfully mitigated this drawback by introducing organo-clay. This demonstrates that PAO-based fluids can achieve sedimentation stability comparable to or even superior to that of high-viscosity natural oils.

Based on the above background, this study aims to investigate the influence of different bentonite addition levels on the comprehensive performance of BMRFs using PAO oil as the carrier fluid. In this study, PAO is selected as the carrier fluid. Compared to commonly used mineral oils or aqueous media, PAO offers superior thermal and oxidative stability, along with a wider operational temperature range, making it suitable for demanding magnetorheological applications. The novel aspect of this work lies in investigating the synergistic effect between this high-performance synthetic oil and bentonite, aiming to develop an MRF and systematically evaluate its sedimentation stability, steady-state shear rheological properties, dynamic viscoelasticity, and redispersibility. The findings are expected to clarify the mechanism of bentonite in BMRFs, determine its optimal concentration in PAO-based magnetorheological fluids, and provide guidance for the preparation of high-performance magnetorheological fluids with enhanced stability, superior magnetorheological response, and excellent redispersibility.

## 2. Materials and methods

### 2.1 Preparation of the BMRF

**Fig. 1:**
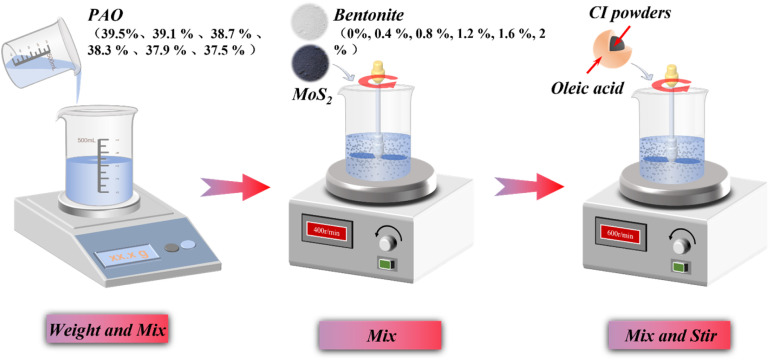
Preparation of BMRF.

The bentonite-modified magnetorheological fluid (BMRF) was formulated with medium-viscosity poly-alpha-olefin (PAO) oil, carbonyl iron powder (CIP), oleic acid, molybdenum disulfide (MoS_2_), and bentonite. Bentonite was introduced as a rheological modifier to enhance the fluid's viscosity and consequently improve its sedimentation stability. MoS_2_ was incorporated as a solid lubricant additive to reduce friction between solid particles and improve the long-term durability of the fluid in dynamic applications. The preparation procedure illustrated in Fig. 1, was as follows. First, PAO oil was weighed using an electronic analytical balance (with successive masses of 39.5 g, 39.1 g, 38.7 g, 38.3 g, 37.9 g, and 37.5 g, corresponding to samples with increasing bentonite content) and placed into a beaker. Subsequently, bentonite (with respective masses of 0 g, 0.4 g, 0.8 g, 1.2 g, 1.6 g, and 2.0 g) and 0.5 g of MoS_2_ were added to the PAO oil. This mixture was then mechanically stirred at 400 rpm for 30 minutes at room temperature to ensure preliminary dispersion. Finally, 60 g of the pretreated CIP compound (CIP pre-coated with oleic acid) was weighed and introduced. The complete mixture then underwent further mechanical stirring at 600 rpm for 2 hours at room temperature to achieve a homogeneous suspension. This process resulted in the preparation of BMRFs with a fixed CIP mass fraction of 60 wt%. The CIP was the EW type from BASF, with a median particle size of 3.0–4.0 µm.

The prepared samples were designated sequentially as BMRF-0, BMRF-4, BMRF-8, BMRF-12, BMRF-16, and BMRF-20, corresponding to the mass of bentonite added. The specific information and detailed formulations of the raw materials used for the BMRF samples are summarized in Table 1.

**Table 1:** Composition of BMRF

Materials	Weight	Weight percentage	Other information
PAO	39.5–37.5 g	39.5–37.5%	PAO-40, TOMA Co., Ltd
CIP compound	60 g	60%	EW, BASF
			Sinopharm Group Chemical Reagent Co., Ltd
MoS_2_	0.5 g	0.5%	Zhenhan New Material Co., Ltd
Bentonite	0–2 g	0–2%	Zhejiang Fenghong New Materials Co., Ltd

### 2.2 VSM test of CIP and BMRF

The magnetic properties of the pure CIP and the representative MRF composite were characterized using a Vibrating Sample Magnetometer (VSM, Model 7404, Lake Shore Cryotronics, Inc., USA). Measurements were performed at room temperature with the applied magnetic field swept between −20 000 Oe and 20 000 Oe. The instrument has a high magnetic moment sensitivity to ensure accurate detection of the samples' magnetization.

### 2.3 Rheological measurement of the BMRF

**Fig. 2:**
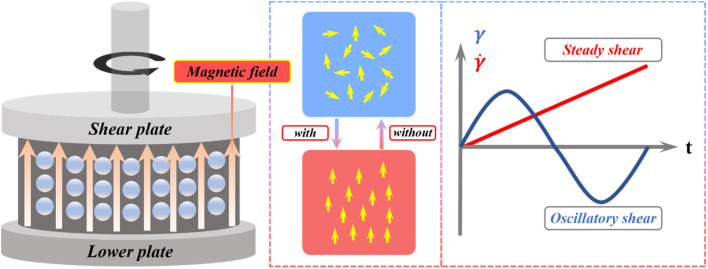
Rheological properties test principle.

Steady-state and oscillatory shear rheological tests were performed on the BMRF samples using a rotational rheometer (Model: Anton Paar MCR302e, Austria) equipped with a standard magnetorheological measurement cell (PS-MRD). This device can apply a maximum current in the range of 0 to 5 A, corresponding to a magnetic flux density of 0 to 1088 mT. An appropriate amount of the sample was loaded onto the rheometer's parallel-plate measuring system, with a fixed gap of 1 mm set between the plates. The working principle of the rheometric measurement is schematically depicted in Fig. 2. All tests were conducted under isothermal conditions maintained at 25 °C.

For the steady-state shear tests, the relationships between viscosity/shear stress and both the applied current (magnetic field strength) and shear rate were investigated for the different BMRF samples, alongside an assessment of performance recovery after sedimentation. The operating principle for the steady-state shear mode is represented by the red line in Fig. 2. During testing, the shear rate was logarithmically swept from 0.01 s^−1^ to 100 s^−1^. The excitation current was varied from 0 A to 2 A in increments of 0.5 A, corresponding to magnetic flux densities ranging from 0 mT to approximately 491 mT. Additional tests were conducted at currents of 3 A and 4 A (corresponding to ∼726 mT and ∼930 mT, respectively) to determine if the BMRFs had reached magnetic saturation.

In the oscillatory tests, the temperature was maintained at room temperature (25 °C). A constant frequency of 1 Hz was applied while the shear strain amplitude was varied from 0.001% to 10%, and measurements were taken at four applied current levels (0 A, 1 A, 2 A, 3 A) to obtain the curves of the material's storage modulus (*G*′) and loss modulus (*G*″) *versus* shear strain.

## 3. Results and discussion

### 3.1 Sedimentation characteristics of BMRF

In conventional MRFs, the primary cause of sedimentation is the density mismatch between magnetic particles and the carrier fluid. This density difference drives the gravitational settling of particles over time, resulting in the formation of a dense sediment layer and a clear supernatant layer. Such phase separation between the base fluid and magnetic particles can severely compromise the fluid's performance, leading to inconsistent rheological behavior. To evaluate the efficacy of bentonite in mitigating this sedimentation issue, static sedimentation observation tests were conducted on the prepared BMRFs with varying concentrations. Specifically, the prepared BMRF samples were hermetically sealed in transparent glass test tubes and allowed to stand undisturbed at room temperature. The sedimentation process was monitored over several weeks by capturing digital photographs of the test tubes at 48 hours intervals. The sedimentation stability was quantitatively assessed by calculating the sedimentation ratio. This ratio was determined by measuring the volume of the clear supernatant (*V*_s_) and the total initial volume of the suspension (*V*_0_) from the captured images. The anti-sedimentation ratio (*S*) was then calculated using eqn (1). A higher anti-sedimentation ratio indicates a more stable suspension with superior resistance to sedimentation.1
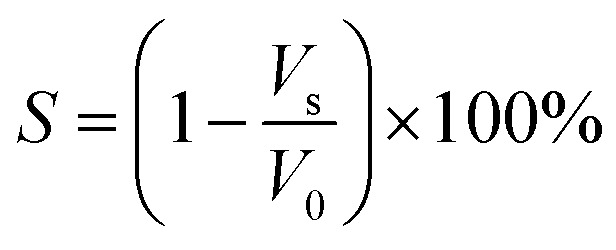
In the equation, *V*_s_ represents the volume of the settled supernatant phase, and *V*_0_ represents the original total volume of the BMRF loaded into the test tube.

**Fig. 3:**
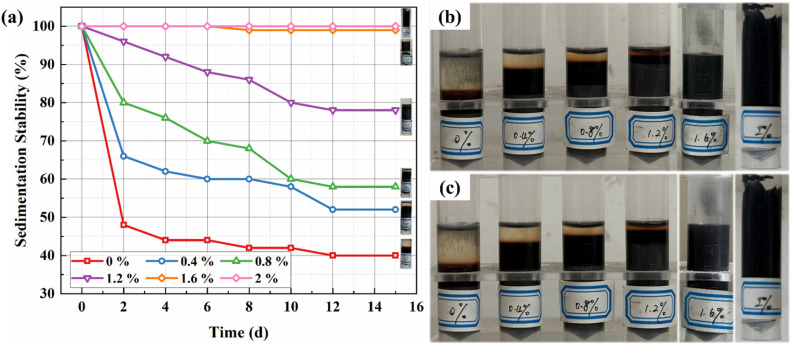
Sedimentation stability of BMRFs. (a) *S*-Value curves; (b) sedimentation status on day 4; (c) sedimentation status on day 12.

Fig. 3 illustrates the anti-sedimentation ratio as a function of time for the BMRFs with different bentonite concentrations (0%, 0.4%, 0.8%, 1.2%, 1.6%, and 2.0%). As observed in the figure, the BMRF samples gradually stratified over time into distinct layers: a clear supernatant layer, an oleic acid separation layer, and a concentrated iron powder sediment layer. The results demonstrate that the sample without bentonite (0%) exhibited the most rapid and severe sedimentation. Its anti-sedimentation ratio, *S*, decreased sharply within the first 48 hours and dropped to approximately 40% by the 12th day. For a MRF with a 60 wt% CIP content, this value indicates near-complete sedimentation, reflecting very poor stability. This suggests that in the absence of any stabilizer, the dense magnetic particles settle rapidly under gravity, forming a dense, hard-packed sediment that is difficult to redisperse.

In contrast, the sedimentation stability of the BMRFs improved markedly with increasing bentonite content. The bentonite-containing samples demonstrated a significantly slower sedimentation rate and achieved a higher final S value. Specifically, the stability enhanced progressively as the bentonite concentration increased from 0.4% to 1.6%. The 1.2% and 1.6% sample exhibited excellent stability, showing the slowest sedimentation throughout the testing period and maintaining an anti-sedimentation ratio of approximately 78% and 99% after 15 days.

This enhancement in anti-sedimentation performance can be attributed to the formation of a three-dimensional network structure within the suspension, induced by the bentonite. As a hydrophilic clay, bentonite swells and exfoliates in the carrier fluid, dispersing into numerous nano- to micro-scale layers. These layers interact with each other *via* van der Waals forces and electrostatic interactions, creating a space-filling, gel-like matrix that markedly improves stability. At a lower bentonite content (0.4%), a preliminary and relatively weak network begins to form, resulting in a modest improvement in stability. As the concentration increases to 1.2%, the network becomes more continuous and robust, leading to a dramatic enhancement in stability.

However, the 2.0% bentonite concentration fundamentally altered the material's characteristics, transforming the MRF into a complete paste that lost all fluidity. Meanwhile, the sample with 1.6% also exhibited slight caking. When inverted, no flow was observed, and the paste-like material could not be readily discharged from the test tube. This is because the excessively high concentration of bentonite led to the formation of an overly rigid and continuous network structure. This resulted in a material lacking free-flowing characteristics, resembling a solid-like gel or paste. Although its measured anti-sedimentation ratio would theoretically be the highest, this state is undesirable for most MRF applications, which typically require the fluid to flow freely and be easily redispersible after sedimentation to restore its original performance.

### 3.2 Magnetic properties of CIP and BMRF composite

**Fig. 4:**
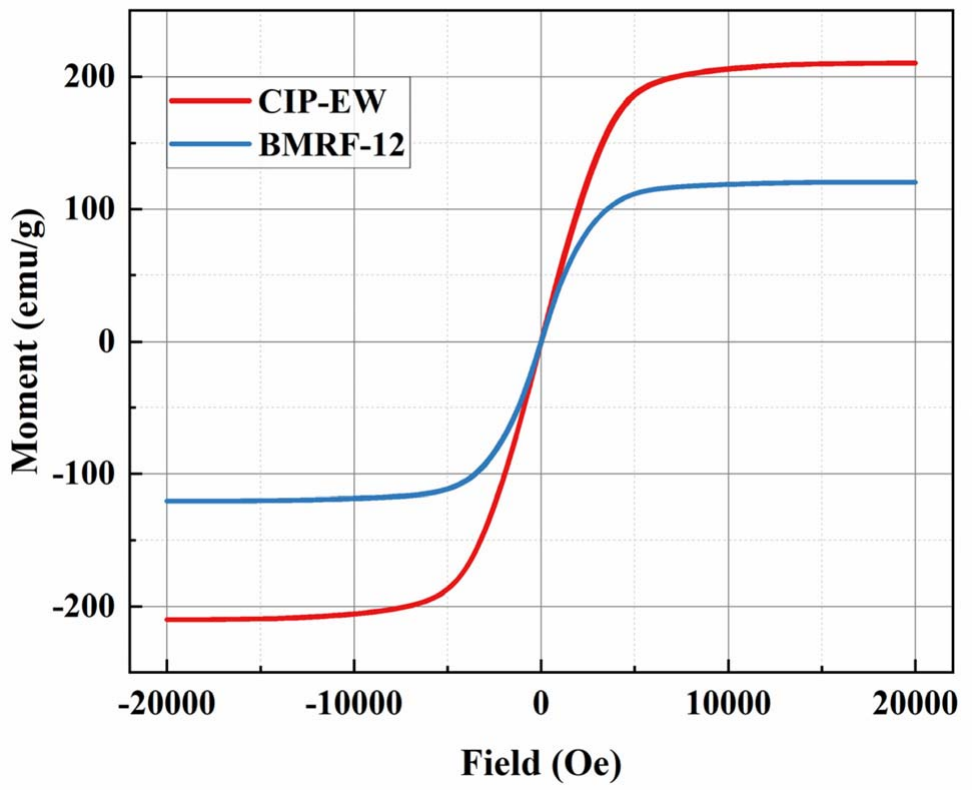
Room-temperature hysteresis loops of the pure CIP (EW) and the BMRF-12.

Fig. 4 presents the room-temperature hysteresis loops for the pure EW-type carbonyl iron powder (CIP-EW) and the BMRF with 1.2% bentonite (BMRF-12). Both samples exhibit typical soft magnetic characteristics, characterized by extremely narrow hysteresis loops, very low coercivity, and negligible remanence. This property ensures that the particles/fluid do not retain significant residual magnetism after the removal of an external magnetic field, facilitating easy control.

Analyzing the magnetization curves, the saturation magnetization (*M*_s_) of the pure CIP is approximately 209 emu g^−1^. In contrast, the *M*_s_ value for the BMRF-12 composite decreases to 119 emu g^−1^. This reduction is primarily attributed to the volumetric dilution effect caused by the incorporation of non-magnetic components (bentonite and MoS_2_) into the composite structure. Despite this decrease in magnetization, the composite retains a sufficiently high magnetic responsiveness to generate the required yield stress for magnetorheological applications.

Moreover, the magnetization curve of the pure CIP tends to saturate at an applied field of approximately 8000 Oe (636.6 kA m^−1^), while that of the BMRF-12 composite saturates at a lower field of about 5000 Oe (397.8 kA m^−1^). Considering that the magnetic field generator of our rheometer can produce fields of approximately 391.0 kA m^−1^ and 662.6 kA m^−1^ at applied currents of 2 A and 3.5 A, respectively, it is confirmed that the magnetic particles are in a fully magnetized state under the high-current conditions employed in rheological tests.

### 3.3 Rheological properties of BMRF

#### 3.3.1 Steady shear rheological properties of BMRF

**Fig. 5:**
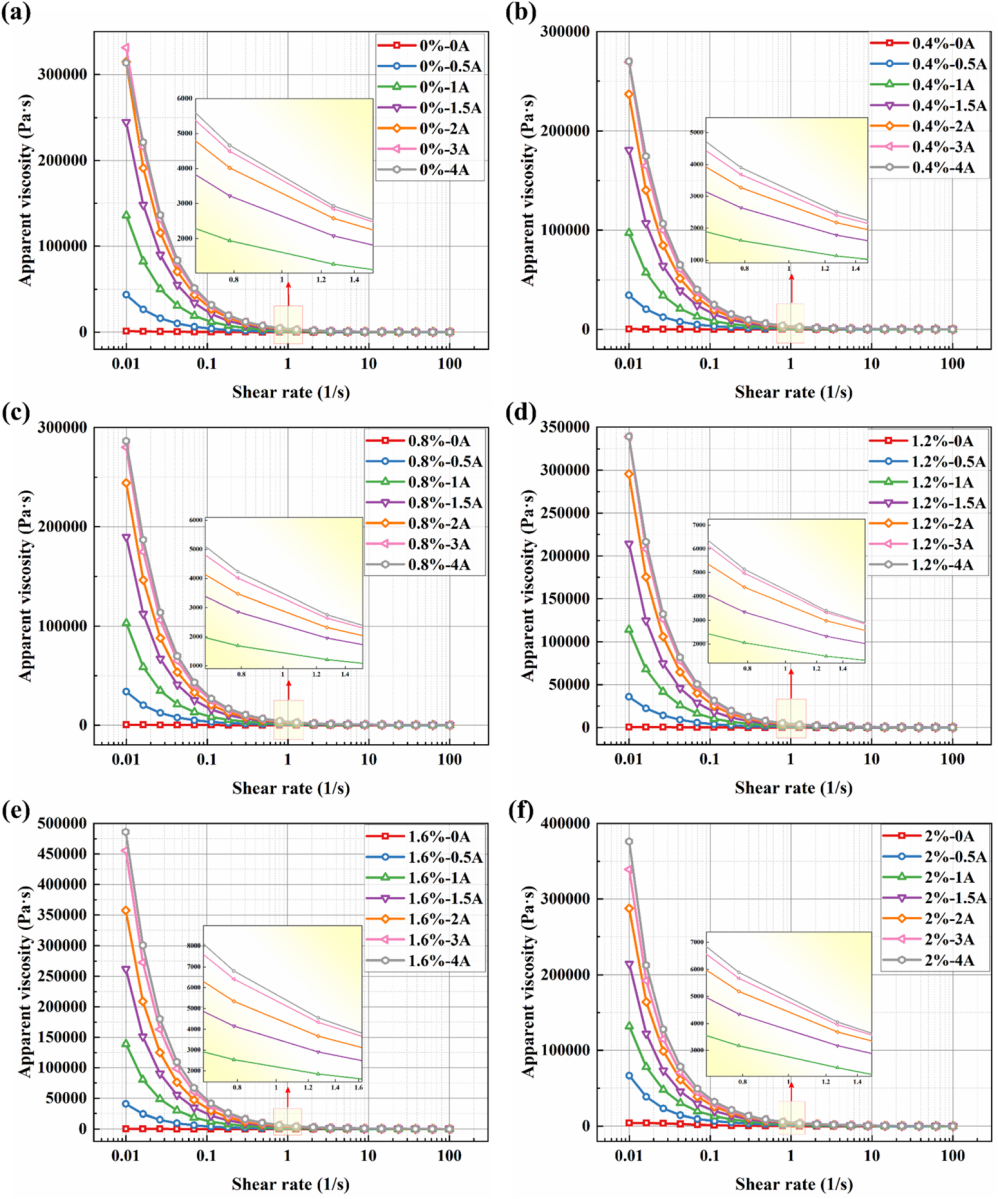
Relationships between apparent viscosity and shear rate for BMRFs with different bentonite concentrations under various applied currents: (a) 0%, (b) 0.4%, (c) 0.8%, (d) 1.2%, (e) 1.6%, and (f) 2.0%.

The steady-state shear rheological characteristics are fundamental for evaluating the performance of magnetorheological fluids (MRFs) in practical applications, as they directly determine the fluid's behavior under continuous flow. Apparent viscosity is one of the key properties to be considered in the device application of magnetorheological materials. Fig. 5 presents the viscosity curves of the BMRFs as a function of shear rate under different applied currents in steady shear mode. As can be observed from the figure, the viscosity of all BMRF samples increases with rising current, as shown in Fig. 5(a)–(f). Simultaneously, pronounced shear-thinning behavior is evident under both field-on and field-off conditions. As the current increases gradually from 0 A to 4 A, the magnetorheological effect becomes more substantial, and the materials demonstrate higher viscosity levels.

**Fig. 6:**
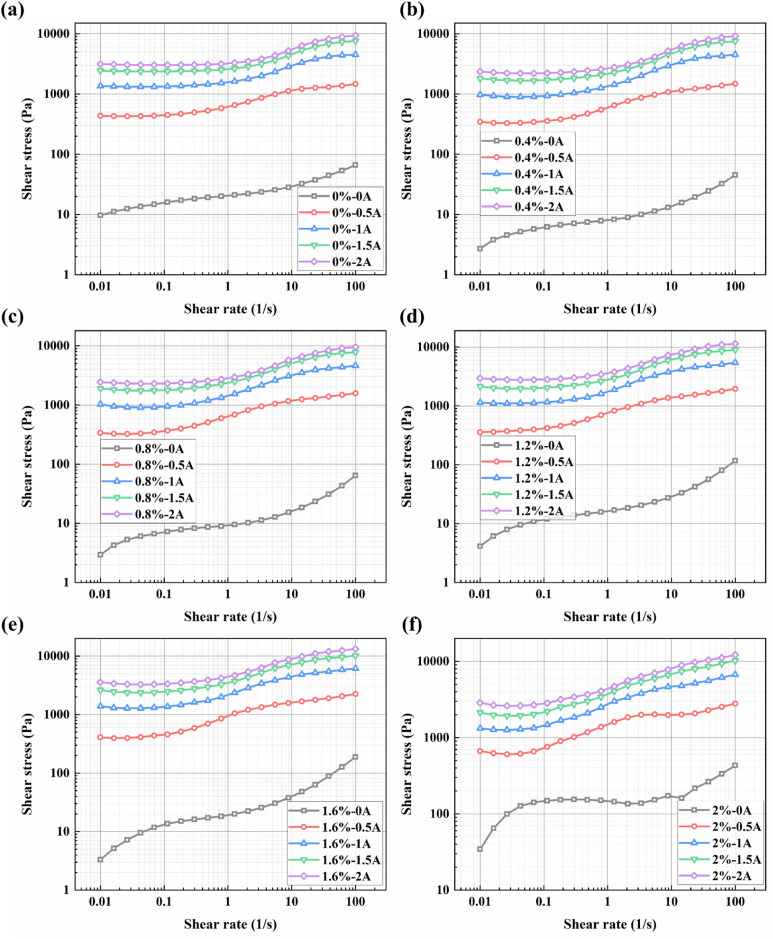
Relationships between shear stress and shear rate for BMRFs with different bentonite concentrations under various applied currents: (a) 0%, (b) 0.4%, (c) 0.8%, (d) 1.2%, (e) 1.2%, and (f) 2.0%.

Furthermore, the variation trends of shear stress with shear rate for the BMRFs under different applied currents are generally consistent, as shown in Fig. 6(a)–(f). At low shear rates, the incomplete yielding of the material leads to a rapid increase in shear stress. As the shear rate further increases, the magnetorheological material progressively yields completely, and the growth trend of shear stress stabilizes. The smoother transition observed in the shear rate range of 0.01 s^−1^ to 10 s^−1^ with increasing current can be attributed to the continuous evolution of the internal microstructure from an elastic solid-like state to a plastic flow state. At very low shear, the magnetic chains undergo elastic deformation (stretching). As shear increases, the chains begin to fracture and reform (plastic flow). The smoothness of the curve indicates that this process is not a brittle rupture. This behavior is further facilitated by the added bentonite gel network, which provides a buffering effect.

**Fig. 7:**
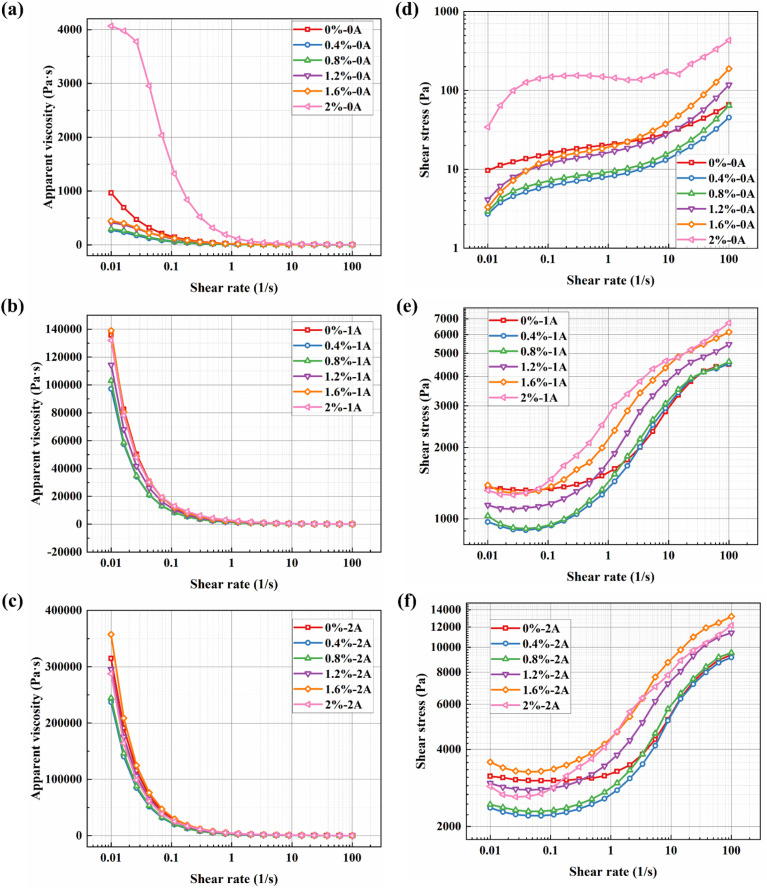
Apparent viscosity and shear stress *versus* shear rate curves under different applied currents: (a) 0 A, (b) 1 A, (c) 2 A, (d) 0 A, (e) 1 A, and (f) 2 A.

The relationship between viscosity and shear rate for different BMRF concentrations under various applied currents is shown in Fig. 7(a)–(c). It can be observed that under zero-field conditions, when the mass fraction of bentonite is below 1.6%, the viscosity curves largely overlap in the logarithmic coordinate system. However, when the addition amount reaches 2.0%, the material's properties undergo a significant change, characterized by a sudden surge in apparent viscosity, which is consistent with the altered material state observed in the sedimentation experiments. Meanwhile, the relationship between shear stress and shear rate for different BMRF concentrations under various currents is illustrated in Fig. 7(d)–(f). Overall, influenced by the magnetorheological effect, the shear stress under zero-field conditions is primarily determined by the base material, namely PAO oil. With the addition of bentonite, the increased material viscosity leads to a corresponding rise in shear stress. As mentioned previously, when the bentonite content reaches 2.0%, the material's properties change, resulting in a sharp increase in shear stress. When the current intensity increases, the carbonyl iron powder particles form magnetic chains aligned with the direction of the applied magnetic field. The strength of these magnetic chains determines the material's shear stress and concurrently increases the viscosity.

To further investigate the relationship between the material's yield stress and the applied current, the steady-state shear flow curves of all BMRF samples under various applied currents, as shown in Fig. 5, were fitted using the Bingham model.^25^ This model is widely employed to describe the behavior of yield-stress fluids:

2 *τ* = *τ*_y_ + *η*_0_*γ̇*

<svg xmlns="http://www.w3.org/2000/svg" version="1.0" width="10.615385pt" height="16.000000pt" viewBox="0 0 10.615385 16.000000" preserveAspectRatio="xMidYMid meet"><metadata>
Created by potrace 1.16, written by Peter Selinger 2001-2019
</metadata><g transform="translate(1.000000,15.000000) scale(0.013462,-0.013462)" fill="currentColor" stroke="none"><path d="M320 960 l0 -80 80 0 80 0 0 80 0 80 -80 0 -80 0 0 -80z M160 760 l0 -40 -40 0 -40 0 0 -40 0 -40 40 0 40 0 0 40 0 40 40 0 40 0 0 -280 0 -280 -40 0 -40 0 0 -80 0 -80 40 0 40 0 0 80 0 80 40 0 40 0 0 80 0 80 40 0 40 0 0 40 0 40 40 0 40 0 0 80 0 80 40 0 40 0 0 120 0 120 -40 0 -40 0 0 -120 0 -120 -40 0 -40 0 0 -80 0 -80 -40 0 -40 0 0 200 0 200 -80 0 -80 0 0 -40z"/></g></svg>


*

where *τ* is the shear stress, *τ*_y_ is the yield stress, *η*_0_ is the plastic viscosity (the constant slope of the shear stress *vs.* shear rate curve after yielding), and *γ̇* is the shear rate.

To quantitatively evaluate the rheological characteristics presented in Fig. 5 and Table 2 shows the fitting results of the rheological data using the Bingham model. The table reveals that, under zero-field conditions, the control sample without bentonite exhibited an anomalously high apparent yield stress. This phenomenon likely occurs because severe sedimentation formed a dense sediment layer, and the measured yield stress essentially represents the stress required to break apart this solid-like deposit. This observation further confirms the critical role of the bentonite network in maintaining a homogeneous suspension and providing a truly reversible, field-controlled yield stress.

**Table 2:** Bingham model parameters fitted from steady-state flow curves at different applied currents

Concentration	Current and parameters					
	0 A		1 A		2 A	
	*τ*_y_	*η*_0_	*τ*_y_	*η*_0_	*τ*_y_	*η*_0_
0%	26.99	0.41	3190.15	15.97	5897.48	40.64
0.4%	10.74	0.35	3265.30	14.82	5817.28	38.93
0.8%	10.79	0.53	3345.64	14.53	6244.03	38.57
1.2%	19.08	0.99	3979.49	16.43	7782.51	42.65
1.6%	24.20	1.65	4566.31	17.65	9369.95	44.02
2%	140.48	2.99	4575.71	22.83	8264.82	42.93

In the presence of a magnetic field, the yield stress of the materials overall shows an increasing trend with rising current. Furthermore, the fitted plastic viscosity also increases with the applied current. Under a magnetic field, the medium flowing in the post-yield stage is not the Newtonian base fluid but contains “magnetic particle clusters” generated by the fragmentation of field-induced chains. A stronger magnetic field produces more robust initial chains, which subsequently break into larger and more persistent flowing clusters, thereby increasing the viscous resistance after yielding.

With increasing bentonite content, the yield stress of the 2.0 wt% sample is significantly higher than that of the other samples. For the samples whose fundamental state did not undergo a direct change, both the yield stress and plastic viscosity increase with concentration.

**Fig. 8:**
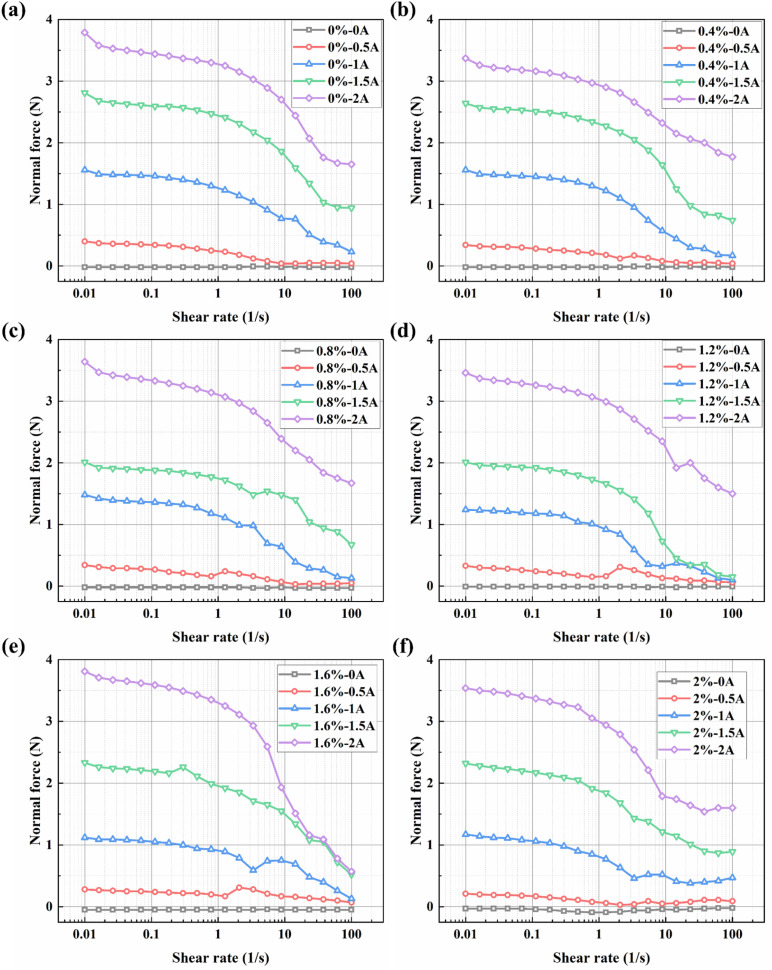
Normal force *versus* shear rate curves for BMRFs with different concentrations: (a) 0%, (b) 0.4%, (c) 0.8%, (d) 1.2%, (e) 1.6%, and (f) 2.0%.

Fig. 8(a)–(f) show the curves of normal force *versus* shear rate for different samples under various applied currents. The relationship between normal force and shear rate for all samples with different bentonite concentrations can be described in two distinct stages. In the low shear rate region, the normal force exhibits a stable plateau. Particularly under higher currents (*e.g.*, 1.5 A and 2 A), the force value remains relatively constant or shows an extremely slow decline. This is because, at low shear rates, the chain-like or columnar structures formed by magnetic particles along the magnetic field direction undergo only tilting and stretching without fracture when subjected to shear. The normal force in this stage is generated by these structures, as the particle chains attempt to expand in the vertical direction during shear, thereby exerting a thrust on the measuring plates.

In the high shear rate region, the normal force shows a decreasing trend. When the shear rate reaches approximately 10 s^−1^, the force value decays significantly. This is because the viscous drag force generated at high shear rates disrupts the magnetic particle chains faster than they can reform. The microstructure collapses into smaller aggregates or individual particles, leading to a reduction in the structural “stiffness” and the associated dilatory effect, consequently causing the normal force to attenuate.

Overall, a strong positive correlation exists between the applied current and the normal force. Under zero-field conditions, due to the absence of particle chains, the normal force is almost negligible. This confirms that in the absence of a magnetic field, the fluid behaves as an ordinary Newtonian fluid without significant elastic microstructure to generate normal stress.

#### 3.3.2 Oscillatory shear rheological properties of BMRF

Under oscillatory shear mode, the variations of the storage modulus (*G*′) and loss modulus (*G*″) of the BMRFs with strain amplitude were investigated to analyze the influence of applied current on their dynamic viscoelastic properties. In dynamic viscoelastic analysis, the storage modulus represents the material's ability to store deformation energy and is an indicator of its elastic recovery after deformation. The loss modulus, in contrast, describes the dissipation of energy as heat during deformation, reflecting the viscous character of the material.

**Fig. 9:**
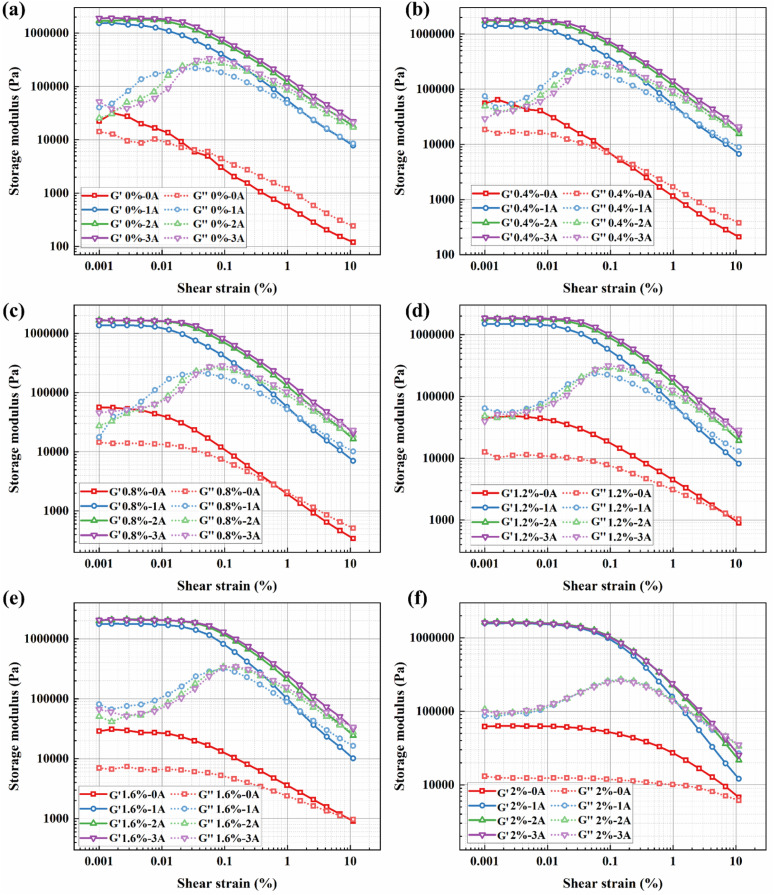
Storage modulus (*G*′)/loss modulus (*G*″) *versus* shear strain curves for BMRFs with different bentonite concentrations: (a) 0%, (b) 0.4%, (c) 0.8%, (d) 1.2%, (e) 1.6%, and (f) 2.0%.

Fig. 9(a)–(f) show the curves of storage modulus *versus* strain amplitude (ranging from 0.001% to 10%) for BMRFs with different concentrations under various applied currents in oscillatory shear mode. It can be seen from the figures that the storage modulus of the BMRFs increases with increasing current, which also indicates that their elastic characteristics become more pronounced. The storage modulus curves of all prepared BMRFs exhibit a plateau region, that is, a region where the storage modulus does not change with shear strain. This plateau region is called the linear viscoelastic region (LVE), which reflects the elastic strength of the material. As the current increases, the linear viscoelastic region expands; simultaneously, as the bentonite concentration increases, the linear viscoelastic region also increases, as shown in Fig. 7(f).

**Fig. 10:**
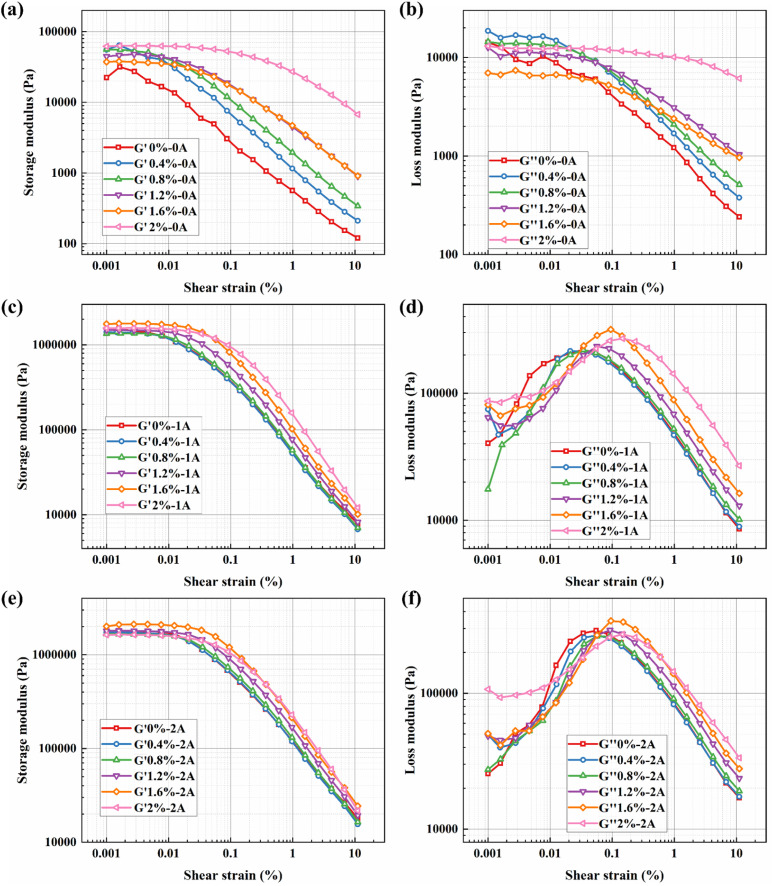
Storage modulus (*G*′)/loss modulus (*G*″) *versus* shear strain curves for BMRFs under different applied currents: (a) *G*′ at 0 A, (b) *G*″ at 0 A, (c) *G*′ at 1 A, (d) *G*″ at 1 A, (e) *G*′ at 2 A, and (f) *G*″ at 2 A.

Furthermore, as observed in Fig. 10(a)–(f), under zero-field conditions, the system lacks long-range strong interactions. The addition of bentonite primarily plays the role of a ‘dispersant’, whose isolating effect dilutes the weak aggregation of CIP particles, leading to a decrease in the plateau modulus with increasing concentration until its own network becomes dominant only at very high concentrations. In the presence of a magnetic field, CIP magnetic chains form within the BMRF, becoming the primary source of stiffness. At this stage, low concentrations of bentonite slightly interfere with the perfect formation of magnetic chains, causing an initial decrease in modulus. However, when the bentonite concentration is sufficiently high, the resulting continuous elastic network strongly couples with the magnetic chains: the network not only stabilizes the particles and promotes homogenization of the magnetic chains but also causes the modulus to rise significantly in the higher concentration range.

At large strains (*e.g.*, *γ* = 10%), regardless of the presence of a magnetic field, transient or fragile structures have already yielded. The residual elasticity of the material is almost entirely determined by the strength and integrity of the permanent physical network formed by bentonite. Consequently, the monotonic increase in storage modulus with bentonite concentration becomes clear and consistent at this stage.

Outside the linear viscoelastic region, the curve of the BMRF storage modulus *versus* strain amplitude shows a decreasing trend, exhibiting the Payne effect.^26^ The reason is that when the strain amplitude increases, the chain-like structures formed by the magnetic particles are destroyed, and the storage modulus continuously decreases with increasing strain amplitude.

As for loss modulus, it can be observed from the figures that higher currents result in higher peak values of the loss modulus curves, indicating enhanced viscous characteristics of the BMRFs with increasing current. When the strain amplitude is small, significant fluctuations in the curves are evident. This phenomenon is attributed to the complex contact conditions within the internal irregular network structure formed between the bentonite-containing PAO oil matrix and the carbonyl iron particles under a magnetic field. When the strain amplitude becomes larger, the loss modulus of the BMRFs decreases with increasing strain amplitude, and the curves become smoother.

To further elucidate the solidification and structural integrity of the BMRF systems, it is instructive to analyze the viscoelastic moduli crossover points and the damping factor. The crossover point typically marks the transition from a predominantly elastic, solid-like state to a viscous, liquid-like behavior under increasing strain.

For the sample with 2%, the storage modulus remains consistently higher than the loss modulus throughout the entire amplitude sweep. This indicates that the material maintains a stable, solid-like gel structure even under strain, suggesting the formation of a robust percolated network. This observation is consistent with the strong inter-particle interactions described in the analysis of the Payne effect; the filler network is sufficiently developed to resist the transition to a liquid state within the tested strain range.

In contrast, the other samples exhibit a narrower elastic region or a lack of distinct solid-like dominance. In these cases, the premature crossover point, suggests a weaker internal network that is more susceptible to structural breakdown and liquefaction when subjected to deformation.

#### 3.3.3 Effects of different CIP particle sizes and concentrations on convection properties

To further investigate the effects of carbonyl iron powder particle size and concentration on the rheological properties of the material, we introduced particles of different sizes: the OM type from BASF (particle size: 3.9–5.2 µm) and the CN type (particle size: 6.5–8.0 µm). Together with the EW type used in sample preparation (particle size: 3–4 µm), they form a gradient, facilitating the observation of particle size effects on the material's rheological performance. Meanwhile, while keeping the content of other materials constant, the CIP concentration was successively reduced by 10% and 20%.

**Fig. 11:**
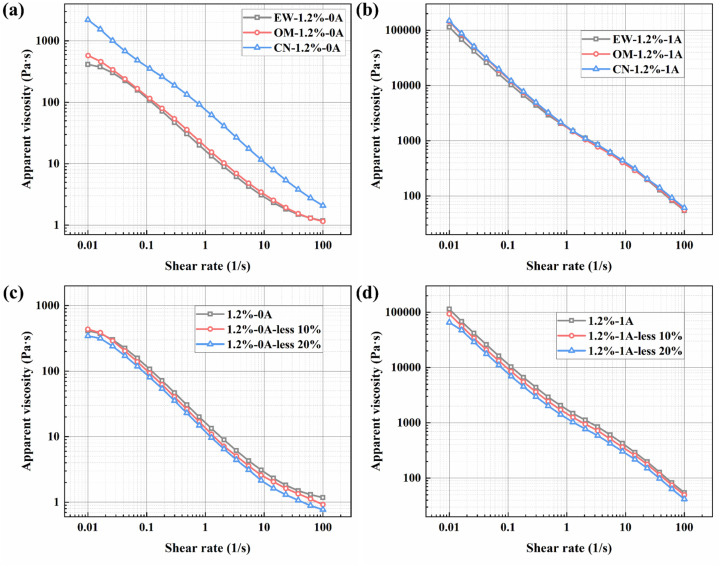
Influence of CIP particle size and concentration on rheological properties (apparent viscosity *versus* shear rate): (a) different CIP particle sizes at 0 A; (b) different CIP particle sizes at 1 A; (c) different CIP concentrations at 0 A; (d) different CIP concentrations at 1 A.

Fig. 11(a) and (b) show the apparent viscosity *versus* shear rate curves for different CIP particle sizes under zero-field and 1 A current conditions, respectively. It can be observed that the influence of different CIP particle sizes on the overall material performance is mainly reflected in the magnitude of apparent viscosity. When the particle size increases beyond a certain critical value, it can significantly alter the apparent viscosity of the material under zero-field conditions, as shown in Fig. 11(a). This is because, in magnetorheological fluids, when the volume fraction of spherical particles approaches or exceeds a critical point, the viscosity of the system increases sharply, making flow difficult. Larger particles require fewer particles to reach effective “jamming,” but more importantly, the gaps between larger particles are also bigger. However, additives like bentonite in the formulation fill the gaps between the large CIP particles. This “binary particle system” greatly increases the effective packing volume fraction of the system, more easily triggering jamming and leading to a sudden increase in zero-field viscosity. However, under an applied current, the influence of particle size on the material is relatively minor, as shown in Fig. 11(b). Overall, as the particle size increases, the apparent viscosity of the material under the same conditions increases, meaning it exhibits higher shear stress.

Fig. 11(c) and (d) show the apparent viscosity *versus* shear rate curves for different CIP concentrations under zero-field and 1 A current conditions, respectively. The figures indicate that as the concentration decreases, the apparent viscosity of the material also decreases. In the presence of a magnetic field, this decreasing trend is more pronounced compared to the effect of particle size, suggesting that the CIP concentration affects the upper limit of shear stress.

**Fig. 12:**
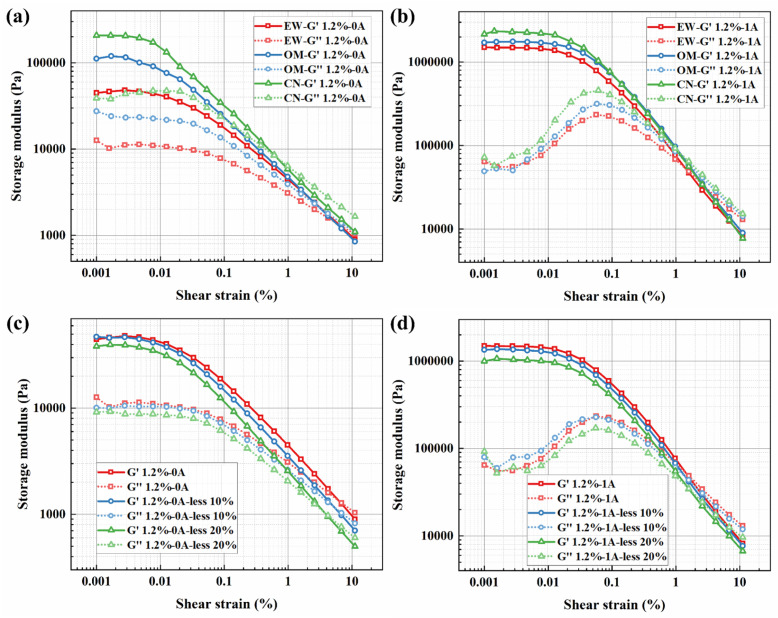
Influence of CIP particle size and concentration on rheological properties (storage modulus/loss modulus *versus* shear strain): (a) different CIP particle sizes at 0 A; (b) different CIP particle sizes at 1 A; (c) different CIP concentrations at 0 A; (d) different CIP concentrations at 1 A.

From Fig. 12(a) and (b), it can be seen that as the CIP particle size increases, both the storage modulus and loss modulus of the material increase, and the crossover point of the storage and loss modulus curves shifts forward. This indicates that the magnetization chains formed by larger particle structures can enhance the elastic response at small strains. However, such coarse structures are more prone to fracture, causing significant energy dissipation and leading to a higher peak in the loss modulus. The forward shift of the crossover point indicates that the material can maintain solid-like behavior at very small strains but rapidly transitions to liquid-dominated flow once it yields. From Fig. 12(c) and (d), it is evident that under both zero-field and 1 A applied current, when the CIP concentration is reduced by 10% and 20%, both the storage and loss moduli of the samples decrease across the entire strain sweep range. Furthermore, the peak of the loss modulus also decreases with reduced concentration. This demonstrates that CIP concentration is a decisive factor for the material's structural strength and viscoelasticity; reducing the concentration weakens the magnetorheological effect and the supportive role of the network.

### 3.4 Redispersibility of BMRF

#### 3.4.1 Apparent viscosity

In addition to static sedimentation stability, the redispersibility of magnetorheological fluids—defined as the ease with which settled sediment can be rehomogenized to restore the fluid's original properties—is critical for their cyclic usability and long-term reliability in practical applications. Fluids that form hard, irreversible sediment cakes during prolonged storage are practically unusable. Therefore, after the static sedimentation tests, the samples were subjected to a redispersion procedure *via* manual shaking and mechanical stirring. The redispersibility of the BMRFs was subsequently evaluated by repeating the steady-state and oscillatory shear tests described in Section 3.2. The redispersion performance was quantified by the percentage change in key parameters such as viscosity and storage modulus before and after redispersion.

**Fig. 13:**
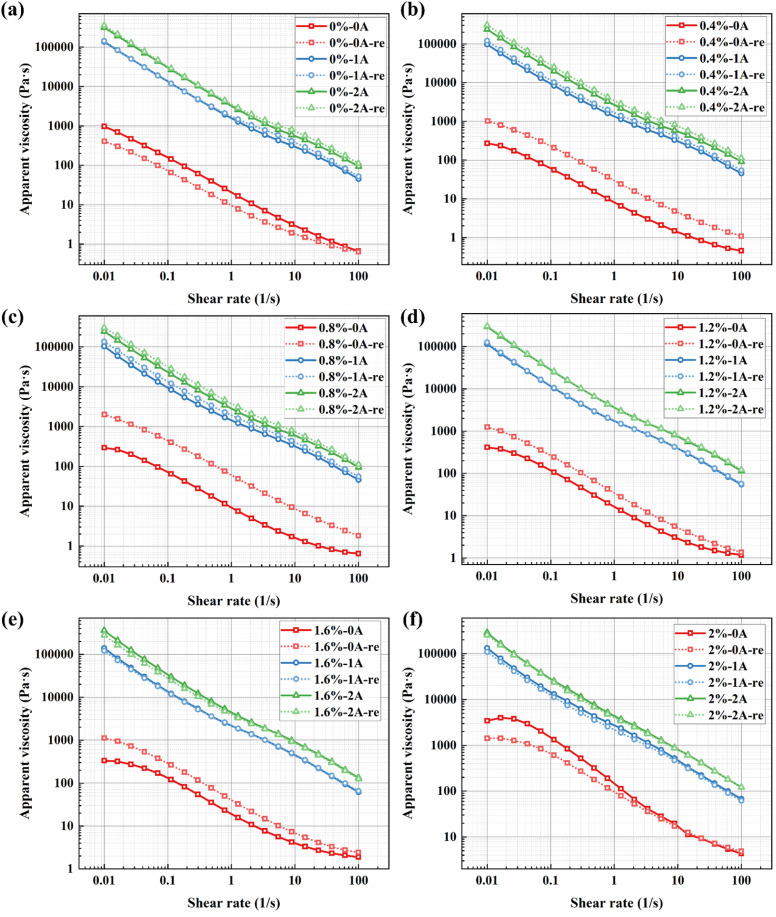
Analysis of the redispersibility for BMRFs with different concentrations under various currents, taking apparent viscosity *versus* shear rate as an example: (a) 0%, (b) 0.4%, (c) 0.8%, (d) 1.2%, (e) 1.6%, and (f) 2.0%.

At a fixed shear rate of 8.86 s^−1^, Fig. 13(a)–(f) illustrate the redispersion performance represented by the apparent viscosity of BMRFs with different concentrations under various applied currents. As can be observed from the figures, the prepared materials exhibit favorable redispersion after simple manual shaking and stirring in the presence of a magnetic field. In contrast, the BMRF without bentonite addition shows poor redispersion recovery under zero field, as seen in Fig. 11(a). Moreover, the sample with 2.0% bentonite, whose material properties have significantly altered, also demonstrates inferior redispersibility.

Table 3 summarizes the apparent viscosity data before and after redispersion at a shear rate of 8.86 s^−1^. A negative percentage change indicates that the material failed to recover its original performance, whereas a positive value suggests that the redispersed fluid exceeds its initial performance. It can be observed that all samples except BMRF-20 exhibit an increase in apparent viscosity after redispersion. Overall, BMRF-12 and BMRF-16 demonstrate relatively stable behavior following redispersion. If excessive bentonite is added, the fluid may undergo property degradation over time, thereby altering its flow behavior, as seen in BMRF-20. It is noteworthy that the variation in zero-field viscosity is the most significant, which could lead to instability under zero-field conditions. However, after the application of current, BMRF-12 maintains the best stability.

**Table 3:** Comparison of the apparent viscosity of BMRFs before and after redispersion at a shear rate of 8.86 s^−1^

Concentration (%)		Current (A) and variation (%)					
		0 A		1 A		2 A	
0%	Initial	3.19	−39%	319.97	29%	589.76	36%
	Redispersion	1.94		413.3		803.08	
0.4%	Initial	1.48	227%	331.23	28%	584.08	42%
	Redispersion	4.84		422.95		828.13	
0.8%	Initial	1.73	446%	346.61	26%	648.53	25%
	Redispersion	9.46		438.15		812.2	
1.2%	Initial	3.10	82%	424.64	1%	815.50	3.7%
	Redispersion	5.64		428.54		846.18	
1.6%	Initial	4.24	73%	490.25	2.5%	985.34	−7%
	Redispersion	7.34		502.48		920.67	
2%	Initial	19.49	−11%	523.98	−11%	880.49	−2%
	Redispersion	17.35		468.41		861.23	

**Fig. 14:**
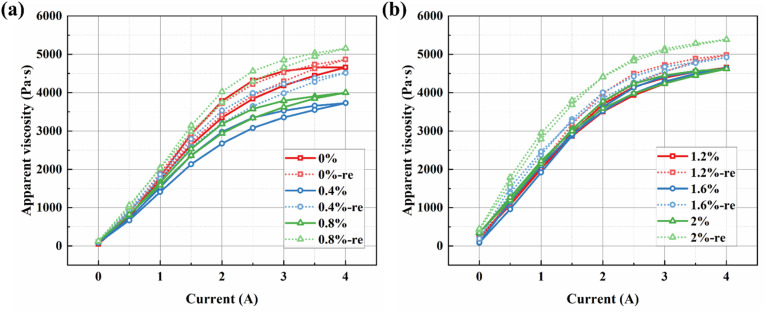
Hysteresis behavior of apparent viscosity during current sweeps for the BMRF samples: (a) 0% to 0.8%, (b) 1.2% to 2.0%.

Fig. 14 presents the relationship between apparent viscosity and the applied current. Since a linear correlation exists between apparent viscosity and shear stress, only the variation in apparent viscosity is analyzed here. Fig. 14(a) shows the data for samples BMRF-0, BMRF-4, and BMRF-8, while Fig. 14(b) presents the data for samples BMRF-12, BMRF-16, and BMRF-20. The hysteresis behavior observed in the current sweeps for all samples indicates an enhancement in the apparent viscosity. This suggests the persistence of some residual particle alignment or internal structure after the magnetic field is removed, which aligns with the subsequent finding of enhanced moduli after sedimentation and redispersion.

#### 3.4.2 Storage modulus

**Fig. 15:**
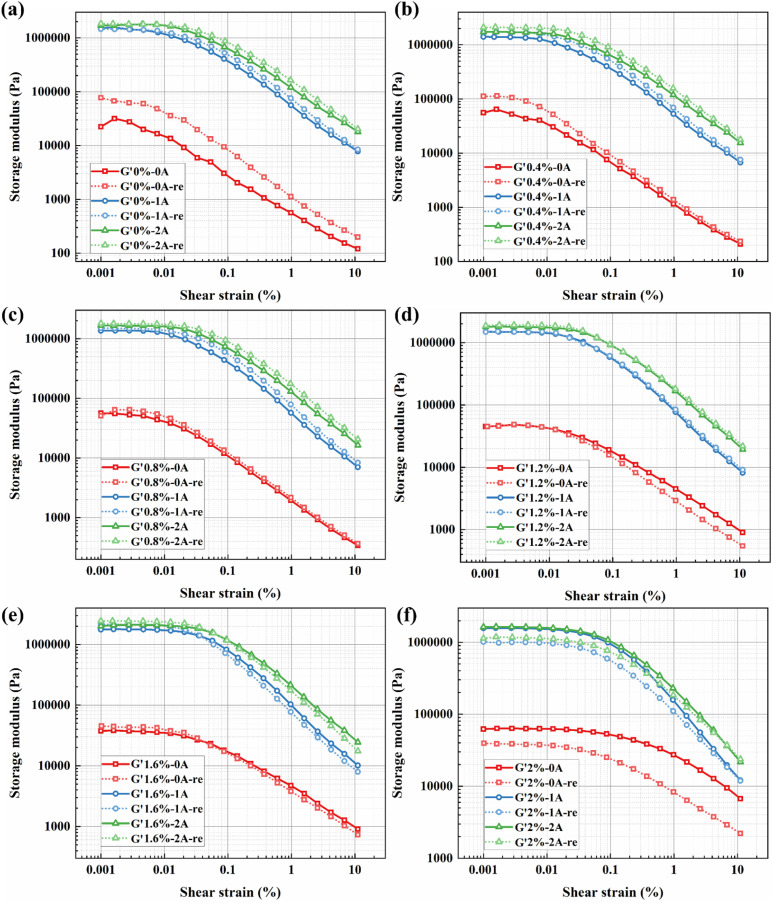
Analysis of the redispersibility for BMRFs with different concentrations under various currents, taking storage modulus *versus* shear strain as an example: (a) 0%, (b) 0.4%, (c) 0.8%, (d) 1.2%, (e) 1.6%, and (f) 2.0%.

At a fixed strain amplitude of 0.0125%, data from the linear viscoelastic (LVE) plateau region were selected for comparison. Fig. 15(a)–(f) illustrate the redispersion performance, represented by the storage modulus, of BMRFs with different concentrations under various applied currents. Consistent with previous analysis, the sample exhibiting altered material properties (2.0% bentonite) demonstrates poor redispersion and recovery performance. In contrast, all other prepared BMRFs were effectively restored to their initial state.

Table 4 summarizes the changes in storage modulus before and after redispersion at the specified strain amplitude of 0.0125%. The interpretation of positive and negative percentage changes remains consistent with the previous section. The data indicate that, except for BMRF-20, all samples show an increase in storage modulus after redispersion, with higher growth rates observed at lower concentrations. This suggests a substantial enhancement in the elastic properties of the material, which may be attributed to the formation of a more uniform and robust three-dimensional network structure by the bentonite during the two-week static storage period. This finding further underscores the importance of employing an appropriate bentonite concentration in material formulation. With increasing bentonite addition, the samples begin to stabilize at a concentration of 0.8%, while BMRF-12 exhibits stronger material stability.

**Table 4:** Comparison of the storage modulus of BMRFs before and after redispersion at a shear strain amplitude of 0.0125%

Concentration (%)		Current (A) and variation (%)					
		0 A		1 A		2 A	
0%	Initial	13 540	165%	1 090 300	12%	1 656 300	4%
	Redispersion	35 882		1 223 400		1 722 300	
0.4%	Initial	30 367	70%	1 083 000	32%	1 590 100	26%
	Redispersion	51 708		1 427 700		2 000 700	
0.8%	Initial	38 162	21%	1 154 600	16%	1 581 300	9%
	Redispersion	46 074		1 335 100		1 723 800	
1.2%	Initial	39 960	2%	1 381 400	3.3%	1 729 100	8%
	Redispersion	40 410		1 427 383		1 862 046	
1.6%	Initial	34 148	10%	1 693 100	17%	2 048 800	14%
	Redispersion	37 448		1 976 100		2 343 300	
2%	Initial	62 358	−41%	1 515 800	−36%	1 571 200	−29%
	Redispersion	36 617		962 640		1 111 700	

Based on the data synthesized from Tables 3 and 4, it can be demonstrated that the BMRFs with bentonite concentrations of 0.8% and 1.2% exhibit enhanced material properties after static storage, while retaining clearly observable macroscopic flow characteristics and showing no significant signs of deterioration.

## 4. Conclusion

This study systematically investigated the effects of bentonite concentration on the sedimentation stability, MR properties, and redispersibility of BMRFs. BMRFs were prepared by dispersing carbonyl iron powder (60 wt%) and varying amounts of bentonite (0–2.0 wt%) in a PAO oil base, with oleic acid and molybdenum disulfide as additives. Sedimentation tests revealed that bentonite significantly enhances stability by forming a three-dimensional network structure, which impedes particle settling and improves the anti-sedimentation ratio. The sample with 1.2 wt% bentonite exhibited optimal sedimentation stability, retaining 78% of its initial state after 15 days. However, at 2.0 wt%, the BMRF lost fluidity and transformed into a paste-like solid, while at 1.6 wt%, partial caking (block-like flow behavior) was observed.

Steady and oscillatory shear tests demonstrated that bentonite improves both the MR effect and the viscoelastic properties. Apparent viscosity and shear stress increased with rising magnetic field strength, and the linear viscoelastic region expanded with higher bentonite content. Fitting the shear stress–shear rate curves using the Bingham model further established clear correlations: under a fixed magnetic field, yield stress increased with bentonite content, confirming the reinforcing role of the clay network. The 2.0 wt% sample showed a significantly higher zero-field yield stress, indicating the formation of a percolated solid-like network. For a given bentonite concentration, yield stress increased dramatically and monotonically with applied current, highlighting the dominant and reversible contribution of field-induced particle chains. Notably, bentonite promoted earlier magnetic saturation by facilitating particle pre-alignment within the network. Redispersibility evaluation indicated that the addition of bentonite enabled the BMRFs not only to recover but also to improve their original performance after redispersion, with increases in both apparent viscosity and storage modulus. The 1.2 wt% sample, in particular, demonstrated stronger stability. In contrast, the 2.0 wt% sample exhibited irreversible property degradation.

In summary, bentonite effectively enhances the overall performance of MRFs by acting as a structural modifier and stabilizer. The established quantitative relationships among bentonite concentration, applied current, and key rheological parameters provide a valuable framework for the rational design of high-performance MRFs with balanced stability, strong field-responsive characteristics, and long-term service reliability.

## Conflicts of interest

The authors declare that they have no known competing financial interests or personal relationships that could have appeared to influence the work reported in this paper.

## Supplementary Material

RA-016-D5RA09063F-s001

RA-016-D5RA09063F-s002

## Data Availability

The data supporting this article have been included as part of the supplementary information (SI). Supplementary information is available. See DOI: https://doi.org/10.1039/d5ra09063f.
